# The Antiseptic and Eliminative Treatment of Typhoid Fever

**Published:** 1901-09

**Authors:** T. Virgil Hubbard

**Affiliations:** President Atlanta Society of Medicine; Assistant to Chair of Therapeutics Atlanta College of Physicians and Surgeons


### THE ANTISEPTIC AND ELIMINATIVE TREATMENT OF TYPHOID FEVER.*

BY T. VIRGIL HUBBARD, M.D.,

President Atlanta Society of Medicine; Assistant to Chair of Therapeutics Atlanta College of Physicians and Surgeons.

In presenting this the third consecutive paper on the same subject to this Association, an apology might be necessary but for the fact that the truth can not be repeated too often.

The modern tendency to investigate so closely the habits and

*Read before the Medical Association of Georgia, at Augusta, April, 1901:

pedigree of the different bacteria, it seems to me, is largely supplanting the investigation of the real condition of the patient, and it is a tendency against which the intelligence of the future will cry out in no uncertain terms.

It is generally admitted that we are constantly surrounded by a variety of these disease-producers; even the air we breathe, the water we drink and the earth we tread are but the natural habitats of these micro-organisms. Every human being probably carries within his own person some one of the different varieties of these germs in sufficient number to produce an infection, yet how many human beings go through a comparatively useful career without ever succumbing to their baneful influence. Now what does this teach? It means there are other factors in the production of this disease than the germ itself, and this factor, in my opinion, to a great extent is autointoxication—a practical fact which surgeons have recognized and heeded by a thorough preparation of their patient before an operation. They thoroughly purge the patient and see that his skin, kidneys and liver are acting freely, thus eliminating all of the effete material and chemical products of tissue changes constantly going on in the human organism.

Park in his surgery goes so far as to say that "in addition to purgatives, drugs like salol, charcoal and salicylates should be administered" as intestinal antiseptics. Many uremic accidents supervening after surgical operations may be atoned and the condition made to pass away by internal administration of naphthalene and charcoal.

Putrefaction of intestinal contents, to my mind, affords a most prolific source of autointoxication. If an attempt at intestinal antisepsis is so desirable before surgical operations, how much more so does it become in an acute infectious disease with its principal seat in the bowels. Here all the conditions favor putrefaction, the presence of bacteria, peptone, heat and moisture, with diminished biliary and intestinal secretions, as a result of fever. With the expectant or "do-nothing" plan of treatment and its accompanying constipation and tympanites, the result of putrefaction, the wonder to me is that more patients do not succumb to perforation and hemorrhage—and it certainly speaks volumes for the resisting power of nature.

In a bowel whose walls are already weakened by ulceration, common sense dictates that by allowing it to become filled with putrefying material, distended with gas as a result, we have the most favorable condition for perforation or rupture of the weakened wall of an artery. Compared to danger like this, the insignificant disturbance produced by mild purgation is but as "the roar of a meadow brook to the wild tumult of a mountain stream."

When we stop to think that there is enough poison produced in the human system in a few hours to destroy life if retained, it requires no prophetic vision to predict that the day is near at hand when the general practitioner will abide by the common sense rule of the surgeon and use the alimentary canal for what nature largely intended it—a sewer to drain from the system the toxins, effete material and other obnoxious products.

Nature intended the bowels to act in health, and if constipation is bad in health, it is worse in disease. It is superfluous to repeat here what I have stated in previous papers, and that is, that I know that you cannot render the alimentary canal aseptic by the administration of drugs, but the man who fails to give some one of the intestinal antiseptics in typhoid fever is derelict in his duty; in not using the resources at his command for the benefit of his patient.

Dr. Osler, who has been the most outspoken opponent against this treatment, and maintained that intestinal anticeptics were useless and should not be administered in typhoid, at the same time highly extols Urotropin to disinfect the urine. If you can disinfect an excretion like the urine by administering antiseptics internally, why won't the same drug destroy the micro-organism in the bowel, the blood or any tissue of the body? Why does the drug possess more antiseptic power when in the urine than it would in the blood?

Is it not more rational to say that a drug given internally is more likely to destroy a bacillus in the bowel than to say it will destroy it after being excreted in the urine, and when Dr. Osler recommends Urotropin to disinfect the urine, he contradicts himself in his argument against intestinal antiseptics, and consequently his logic is false.

There are a number of drugs which can be given in harmless doses that will prevent fermentation and putrefaction of the bowel contents, and consequently the formation of gas. Park says: "Man forms in his liver enough poison to kill him in eight hours, and the urine eliminates but one-half." Bile is nine times as poisonous per volume as urine. Does this not show to any unprejudiced mind the importance of keeping the secretions and excretions in an active condition?

We all know that in fever destructive tissue metamorphosis is taking place more rapidly than in health. More poisons are being manufactured and at the same time, as a rule, functional activity of the emunctories—the liver, kidneys and skin—is lessened; consequently we have the abnormal condition of increased production of poison and diminished elimination, and hence the therapeutic indication becomes very plain. If elimination from the system of these poisons is so necessary in health, how much more necessary does it become in a disease produced by a germ which is constantly manufacturing toxins of unknown chemical composition and lethal power, and sending them to every part of the human system through the medium of the circulation and lymphatic current.

The bacteriologist tells us it is not the germ itself which gives rise to the symptoms, but it is the poison produced by the germs (toxins), and this exegesis of the subject is largely borne out by the fact that few germs are found in the blood; consequently some other agent must be the active factor in the production of symptoms.

Horton-Smith, in the Gouldstonian lectures, showed that "in twelve cases of fatal typhoid fever coming to post-mortem examination, the blood in five was sterile; in two it contained typhoid bacilli; in one streptococci and staphylococci; in two the bacillus coli and in two the bacillus coli with proteus." Out of thirty cases, including his own, the typhoid bacilli was found only in nine. These observations certainly prove two conditions which are, first, that the patient may die with typhoid fever and no germs can be found in the blood, and the other, that of the different varieties of germs found in the blood after death in typhoid fever patients, the bacillus of Eberth is found in less than one-third of the cases and other germs of the different varieties are found in two-thirds of the cases. This element of mixed infection is, in my opinion, responsible for the great variation in symptoms in this disease and accounts for that significant fact which we often hear from physicians, that they scarcely meet with two cases presenting just exactly the same clinical aspect. It is also a forecast of the difficulty we will meet with in attempting to get a successful anti-toxin for typhoid.

It remains for the physiologic chemist to supply the missing link and bridge the chasm which now exists between the established bacteriologic science and a rational and scientific therapy. Every new discovery is attended with its accompanying evil, and the glaring one connected with the valuable enunciation of Koch and Lister is the fact that it has resulted in the production of so many therapeutic nihilists. Some of the best men in the profession, in their eagerness to join the procession in the study of these new and fascinating caustive factors of disease, have overlooked the importance of the thorough knowledge of the physiologic and pathologic chemistry of the human system. The time is now ripe for the pendulum to swing in the opposite direction and let us learn not only of the products of the germ but the complex chemical poisons which are constantly being generated in nature's own laboratory, the human system, a thorough knowledge of which the general practitioner is now seeking, and which will result in the solution of some of the intricate problems of modern medical science. In this connection I desire to present a few clinical facts which show the ridiculous absurdity of some modern theories regarding the administration of drugs in infectious diseases. The modern teaching is all to the effect that infectious diseases run a definite and specific course and cannot be influenced by medication. To this oft-repeated error I desire to enter a protest based on clinical experience.

The man who says you cannot favorably influence the lethal tendency of the caustive factors in typhoid fever by medication has simply not had experience with the proper drug. He belongs to that class of medical men who consider it unscientific to storm the domain of a bacillus with a dose of medicine and has practically discarded his old friend the "Materia Medica." He contents himself by sitting down by the placid waters of expectancy and watching the life of his patient slowly but surely ebb away as a result, not only of the ravages of the supposed germ which is causing the disease, but the chemical toxins as a result of tissue metabolism as well.

With this explanation of what I consider to be the real condition of the patient in typhoid fever, we will see how we can best meet the plain indication for elimination. Some of those who have opposed this treatment maintain that the germ is found in the walls of the bowels and lymphatic glands and other tissues of the body; consequently it cannot be reached by any drug. If they will but review their Materia Medica, they will find that mercury is absorbed into the blood and carried to the remotest part of the system, and is found in the secretions and excretions, even the saliva, the semen and the milk from the mammary gland. So it is immaterial in what tissue the germ may be found, whether the roots of the hair, the tips of the fingers, the spleen or lymphatic glands, it cannot eacape the influence of mercury when given internally.

Is mercury not a specific for that disease which is typical of the class of constitutional disease, syphilis? In controlling this disease does not mercury go to every part of the system? I maintain it is more rational to attempt to reach a germ and its products in whatever tissue the fancy of my opponents may dictate by the administration of mercury internally, than it is to reach the same by the application of cold water to the skin, but it is as important to know how to give mercury in typhoid as it is in syphilis. Bad results will follow improper administration in either disease, but that in no sense lessens the therapeutic value of the drug. I have used and advocated mercury because it has given me the best results. I know of no other constitutional remedy that will thoroughly stimulate the activity of the emunctories.

We should not be wedded to any one drug or combination of remedies, but should constantly keep in view the object to be accomplished, that is elimination. Any harmless procedure that will aid in securing elimination is to be commended. I now use in all my cases, normal salt solution per rectum, and I find it the most efficient means of securing prompt action of the skin and kidneys, and I can not too strongly emphasize the importance of these two emunctories, and especially the kidneys, in typhoid. In profound depression, after hemorrhages and in some cases of low muttering delirium, I immediately resort to the subcutaneous administration of salt solution. I direct that the patient be sponged with water at a temperature most agreeable to him, with brisk rubbing of the skin to stimulate the nervous system. In no case have I had to resort to antipyretics of any sort after two or three days of treatment, and in very few cases is it necessary with this treatment to use alcohol in any form.

Some of the patients go through the disease without the necessity of strychnine. Time forbids me going into details in regard to diet, but as a rule they digest and assimilate more with this treatment than any other plan.

I will briefly repeat the treatment which I recommended in a paper read before this Association one year ago, and I have very few additions to make to the same. When called to a case of typhoid fever, I usually commence by giving the patient a capsule of calomel 1-2 grain, guaiacol carbonate 2 grains, podophyllin 1-40 grain, every two hours for twenty-four to forty-eight hours, depending upon the condition of the bowels. I continue this until I have secured four or five intestinal evacuations for two successive days, and then I leave off the calomel and add 1-2 grain of menthol to the guaiacol and podophyllin. If after discontinuing the calomel there is a tendency (as there frequently is) of the bowels to become inactive, I administer a small dose of salts or Hunyadi water in the morning. I always endeavor to secure at least two or more evacuations daily, depending upon the temperature and condition of the bowels. If after three or four days of treatment, the temperature remains high or rises after having remained stationary, I again resort to the calomel as before for twenty-four hours, or less, as necessary, and it invariably reduces the temperature and results in a general improvement in the patient's condition. I continue the administration of guaiacol and menthol throughout the course of the disease.

Guaiacol carbonate, aside from its antiseptic effect in preventing intestinal putrefaction, seems to have some favorable influence over the disease. Its well-known beneficial effect in tuberculosis strengthens the declaration that it would be beneficial in typhoid.

This is the treatment as I use it in the majority of cases, but I wish it distinctly understood that it is not routine, and that the size and dose and frequency of administration must be varied to suit the case, and the physician at the bedside must determine this fact.

Now to the point that typhoid can be influenced by medication. I have, up to the present time, treated thirty-five cases without a death and in every single case the temperature reached normal by the morning of the fourteenth day without the use of antipyretics. Some of them as early as the tenth, eleventh and twelfth days.

In a few cases it never went above normal again; in the majority of them it would rise a degree, or perhaps a degree and a half, in the afternoon, but the convincing fact is, that the disease was always under control by the fourteenth day, and its course and severity mitigated.

These are not theoretical deductions reached by following in the footsteps of some erroneous text-book writer, nor have they fallen from the lips of some college professor whose verbosity far exceeds his logic, but it is indubitable clinical evidence gleaned from the bedside of the patient.

The fact that in these thirty-five cases there developed no complication during the course of the treatment, is sufficient evidence of its efficacy to convince any unprejudiced mind. Hemorrhage only occurred twice, and then very light. In cases where I have been called in consultation, and complications were already present, they speedily passed away under the influence of this treatment.

In conclusion, I will say that the efficacy of this treatment as demonstrated by the clinical results are the rocky shores of truth against which the fleeting and effervescing waves of the narrow-minded and prejudiced will forever beat in vain. It is too well established with the thinking members of the profession, for its solid foundation ever to be underminded by men who use the flimsy argument, "I do not believe in it."

